# Wissenschaftsbetrug und zweifelhafte Publikationspraktiken

**DOI:** 10.1007/s00063-025-01307-3

**Published:** 2025-08-07

**Authors:** Peter Nydahl, Mohamed Chahdi, Pia Goetze, Roland Eßl-Maurer, Carsten Hermes, Andreas Kocks, Anna-Henrikje Seidlein, Susanne Krotsetis

**Affiliations:** 1 Pflegewissenschaft und -entwicklung, Universitätsklinikum Schleswig-Holstein, Arnold-Heller-Str. 40, Haus V40, 24105 Kiel, Deutschland; 2 Klinik für Neurologie, Universitätsklinikum Schleswig-Holstein, Kiel, Deutschland; 3 Klinik für angeborene Herzfehler und Kinderkardiologie, K1-Ethikberaterin im Gesundheitswesen, Universitätsklinikum Schleswig-Holstein, Kiel, Deutschland; 4 Pflegedirektion, Koordination Entwicklung klinische Pflegepraxis, Uniklinikum Salzburg, Paracelsus Medizinische Privatuniversität, Salzburg, Österreich; 5 Hochschule für Angewandte Wissenschaften Hamburg (HAW Hamburg), Hamburg, Deutschland; 6 Akkon-Hochschule für Humanwissenschaften, Berlin, Deutschland; 7 Pflegeforschung, Universitätsklinikum Bonn, Bonn, Deutschland; 8 Pflegedirektion, Netzwerk Pflegeforschung und Praxisentwicklung, Verband der Pflegedirektorinnen und Pflegedirektoren der Universitätskliniken und medizinischen Hochschulen in Deutschland, Berlin, Deutschland; 9 Institut für Ethik und Geschichte der Medizin, Universitätsmedizin Greifswald, Greifswald, Deutschland; 10 Pflegewissenschaft und -entwicklung, Universitätsklinikum Schleswig-Holstein, Lübeck, Deutschland; 11 Institut für Pflegewissenschaft und -praxis, Paracelsus Medizinische Privatuniversität, Salzburg, Österreich

**Keywords:** Forschungsethik, Peer-Review-Standards, Predatory Journals, Wissenschaftliche Integrität, Fake Science, Research ethics, Peer-review standards, Predatory journals, Scientific integrity, Fake Science

## Abstract

Zweifelhafte Publikationspraktiken und Journale stellen eine zunehmende Herausforderung für die wissenschaftliche Integrität dar. Diese Publikationsmodelle werben mit schnellen Publikationszeiten und zeichnen sich durch fehlende Qualitätskontrollen wie Peer-Review-Verfahren, mangelnde Transparenz und hohe Gebühren aus, was die Verbreitung unzuverlässiger Forschungsergebnisse begünstigt. Insbesondere für weniger erfahrene Forschende besteht ein Risiko, unwissentlich in solchen Journalen zu publizieren oder deren Inhalte unkritisch zu nutzen. Dies kann nicht nur individuelle Karrierewege beeinträchtigen, sondern auch die Wissenschaftsgemeinschaft und die gesellschaftliche Wahrnehmung wissenschaftlicher Erkenntnisse negativ beeinflussen. Der Beitrag beschreibt die zentralen Merkmale von sog. Predatory Journals und analysiert deren potenzielle Konsequenzen für die Wissenschaft sowie für die Qualität und Verbreitung von Forschungsergebnissen. Gleichzeitig werden praxisnahe Orientierungshilfen wie Checklisten und etablierte Ressourcen vorgestellt, die Forschenden dabei helfen, unseriöse Publikationsangebote zu erkennen und zu vermeiden. Abschließend wird auf die Bedeutung einer verstärkten Sensibilisierung für diese Problematik hingewiesen, um wissenschaftliche Standards zu sichern und die Verlässlichkeit von Forschung nachhaltig zu gewährleisten.

## Abstract (en)

Questionable publication practices and predatory journals pose an increasing challenge to scientific integrity. These publication models advertise rapid publication times but lack essential quality controls such as peer-review processes, transparency, and charge high fees, which facilitates the dissemination of unreliable research findings. There is an increased risk, particularly for less experienced researchers, of unknowingly publishing in such journals or using their content uncritically. This can negatively impact individual career paths, the scientific community, and public perception of scientific knowledge. The article outlines the key characteristics of predatory journals and analyzes their potential consequences for science, as well as the quality and dissemination of research findings. Practical tools, such as checklists and established resources, are also presented to assist researchers in identifying and avoiding dubious publication offers. Finally, the article emphasizes the importance of raising awareness about this issue to uphold scientific standards and ensure the long-term reliability of research.

Die Veröffentlichung wissenschaftlicher Arbeiten ist essenziell, doch das Prinzip „publish or perish“ fördert auch fragwürdige Praktiken. Predatory Journals – unseriöse Fachzeitschriften, die schnelle Publikationen gegen hohe Gebühren anbieten – untergraben die wissenschaftliche Integrität. Sie verbreiten manipulierte Artikel, die Forschungsergebnisse verzerren und unerfahrene Forschende sowie die Öffentlichkeit täuschen können. Welche Merkmale kennzeichnen diese Journale? Und welche Auswirkungen haben sie auf Wissenschaft und Gesellschaft? Dieser Beitrag beleuchtet die Mechanismen hinter diesem ausbeuterischen Publikationsmodell und gibt Hinweise, wie man sich davor schützen kann.

## Predatory Journals

In den vergangenen Jahren hat die Einreichung und Verbreitung internationaler Publikationen durch die technologischen Entwicklungen, wie z. B. leichterer Zugang zu Datenbanken und Verlagen, aber auch der Einsatz künstlicher Intelligenz, deutlich zugenommen [4]. Auch der Zugang zu wissenschaftlichen Publikationen ist immer einfacher geworden. Wissen – und leider auch falsches und bewusst manipuliertes Wissen – kann heute über das Internet und soziale Medien sehr einfach verbreitet und abgerufen werden [17]. Gleichzeitig besteht für Wissenschaftlerinnen und Wissenschaftler immer noch ein hoher Druck zur Publikation: „publish or perish“ [20]. Das haben sich einige Verlage zunutze gemacht und eine neue Strategie entwickelt: Predatory Publishing. Im Bereich des wissenschaftlichen Publizierens bezeichnet Predatory Publishing – auch als „Write-only-Publizieren“ oder „täuschendes Publizieren“ bekannt – ein ausbeuterisches Geschäftsmodell [17]. Predatory Journals zeichnen sich u. a. durch hohe Publikationsgebühren, fehlende Prüfung von Qualität und Seriosität und schnelle Review- und Publikationsprozesse (gegen Aufpreis weitere Beschleunigung) aus (vgl. Tab. 1; [1]). Diese räuberischen Verlage und Journale geben vor, legitime wissenschaftliche Plattformen zu sein, haben jedoch oft keine oder nur scheinbare wissenschaftliche Integrität.

**Tab. 1:** Merkmale von Wissenschaftsbetrug und zweifelhaften Publikationspraktiken:

*Fehlende Qualitätskontrolle:* kein oder nur oberflächliches Peer-Review-Verfahren von eingereichten Manuskripten
*Aggressive Werbung:* häufige E‑Mails an Wissenschaftlerinnen und Wissenschaftler mit der Aufforderung, Artikel einzureichen
*Fehlende Transparenz:* keine klaren Informationen über Redaktionsmitglieder, Herausgebende oder Geschäftsmodell; oft einhergehend mit versteckten Kosten, die erst nach der Publikation eingefordert werden
*Hohe Publikationsgebühren:* Autorinnen und Autoren zahlen insgesamt erhebliche Gebühren, ohne dafür eine angemessene Dienstleistung zu erhalten
*Gefälschte Indizes:* Behauptungen, in renommierten Datenbanken (z. B. PubMed, Scopus) indexiert zu sein, ohne tatsächlich dort gelistet zu sein, sowie erfundene Impact-Faktoren
*Schnelle Publikationszeiten:* Manuskripte werden zum Teil oft ohne echte Überprüfung innerhalb weniger Tage veröffentlicht
*Perfekte Studien: *oftmals überraschend positive und im Gegensatz zum allgemeinen Wissensstand stehende Ergebnisse, tendenziöse Berichterstattung, fehlende Daten, ungewöhnlich niedrige Rate an Drop-outs, Sicherheitsereignissen und anderen
*Publikationshäufung: *Wenn in kurzer Zeit mehrere, angeblich getrennte Teams von Autorinnen und Autoren über ein Thema ähnliche RCT oder Metasynthesen zur gleichen Zeit herausbringen, könnte es eine „paper mill“ gewesen sein; ist schwer nachzuweisen, aber Zweifel sind angebracht

Beispiele für räuberische Verlage sind u. a. *MDPI, OMICS International* oder *Bentham Open*, zurzeit werden auch *Hindawi* und *Frontiers* diskutiert [1]. Predatory Journals sind keine Seltenheit: Im Jahr 2015 wurden insgesamt über 8000 Predatory Journals mit über 420.000 Artikeln mit ansteigenden Zahlen identifiziert [18]. Bereits 2015 wurden allein 140 Predatory Journals für die Pflege [15] ausgewiesen. Weitere Beispiele umfassen 59 Predatory Journals mit 5610 publizierten Studien im Bereich Rehabilitation im Jahr 2017 [12] und 225 Predatory Journals in der Disziplin Orthopädie im Jahr 2018 [21]. Als herausforderungsvoll zeigt sich bereits an dieser Stelle: Die Zuordnung ist nicht immer auf den ersten Blick zweifelsfrei möglich und die Übergänge zwischen „seriös“ und „räuberisch“ liegen in Details – Negativlisten sind angesichts der Dynamik und z. T. fließenden Übergängen (z. B. angesichts zunehmend hoher Publikationsgebühren) nur bedingt hilfreich.

## Ursachen des Predatory Publishing

Wissenschaftlerinnen und Wissenschaftler veröffentlichen in Predatory Journals aus unterschiedlichen Gründen, die oft mit fehlendem Wissen, Druck oder irreführenden Praktiken zusammenhängen [11]. Die häufigsten Motive und Ursachen sind [20]:

- fehlende Erfahrung oder Wissen (Unkenntnis über Predatory Journals, Vertrauen in irreführende Werbung);
- Druck zur Veröffentlichung („Publish-or-perish“-Mentalität, schnelle Publikation);
- hohe Ablehnungsraten bei seriösen Journalen (mangelnde Qualität der eigenen Forschung, zeitaufwändiger Peer-Review-Prozess);
- Täuschung und Irreführung durch Journale (aggressive Einladungen, Vortäuschung von Legitimität, falsche Versprechen);
- Karriere in Gefahr (fehlende institutionelle Unterstützung, sprachliche Hürden);
- finanzielle Überlegungen (Werbung für zunächst scheinbar geringere Gebühren);
- bewusste Wahl (Verzweiflung oder Opportunismus, Absichten für Publikations- oder Bewerbungsstrategien).

Auch wenn die Gründe vielfältig sein können und eine Publikation in einem Predatory Journal zunächst mit kurzfristigen Vorteilen verbunden sein kann, lassen sich dennoch langfristige Folgen nicht vermeiden.

## Folgen des Predatory Publishing

Die Publikation in Predatory Journals hat negative Konsequenzen für individuelle Forschende, die Wissenschaftsgemeinschaft, aber auch für die Gesellschaft. Wissenschaftliche Arbeiten (auch gewissenhaft durchgeführte Studien), die einmal in Predatory Journals veröffentlicht worden sind, haben oft keinen wissenschaftlichen Wert mehr und können der Karriere der Autorinnen und Autoren schaden. Die Folgen können finanzielle Einbußen und Arbeitsplatzverlust sein. Gefälschte Ergebnisse und manipulative Praktiken führen zu Fehlinformationen, die in der echten Forschung zirkulieren [17]. Gerade für unerfahrene Wissenschaftlerinnen und Wissenschaftler wie auch die Bevölkerung kann Predatory Publishing daher ein hohes Risiko beinhalten, die teilweise falschen oder verzerrten Schlussfolgerungen der Publikationen zu übernehmen [10, 19]. Durch ungenügende kritische Reflexion können diese Aufsätze in eigenen Forschungsarbeiten z. B. der Pflegewissenschaft und Medizin zitiert werden [16, 19]. Artikel aus Predatory Journals können darüber hinaus auch in Newslettern, Vorlesungen oder auch in Metaanalysen zitiert und weitergegeben werden, sodass sie schließlich auch Schlussfolgerungen, Empfehlungen und Leitlinien beeinflussen können [2, 5]. Publikationen in Predatory Journals haben ein hohes Risiko, nach der Publikation zurückgezogen zu werden (www.retractionwatch.com), was die wissenschaftliche Reputation der Autorinnen und Autoren schädigt.

## Wie lassen sich Predatory Journals und zweifelhafte Publikationspraktiken erkennen?

Es gibt bislang keine klare, einheitliche Definition für Predatory Journals. Ein Versuch lautet:

„Predatory Journals und -Verlage sind Einrichtungen, die Eigeninteressen auf Kosten der Wissenschaft in den Vordergrund stellen und sich durch falsche oder irreführende Informationen, Abweichungen von bewährten Redaktions- und Veröffentlichungspraktiken, mangelnde Transparenz und/oder aggressive und wahllose Werbepraktiken auszeichnen (eigene Übersetzung; [9]).“

Die Merkmale sind in Tab. 1 zusammengefasst. Ein weiteres Kennzeichen für Predatory Journals können gefälscht hohe Angaben für Impact Factors sein, die sich auf mithilfe von Clarivate überprüfen lassen (siehe unten: https://jcr.clarivate.com/). Weiterhin haben verschiedene Organisation Checklisten zum Erkennen von Predatory Journals entwickelt, die aber nicht als evidenzbasiert gelten können [14]. Es gilt, sie mit Erfahrung und Sachverstand anzuwenden (Tab. 2). In den letzten Jahren wurden zahlreiche Hilfestellungen und alternative Publikationsmodelle entwickelt.

**Tab. 2:** Checkliste zum Erkennen von Wissenschaftsbetrug und zweifelhaften Publikationspraktiken. (Tabelle in Anlehnung an [1])

*Kennen Sie oder Ihre Kolleginnen und Kollegen die Zeitschrift?*
Eindeutiger, nichtverwechselbarer Name der Zeitschrift?
Neueste Beiträge in der Zeitschrift leicht zu finden?
Abgleich der Informationen über die Zeitschrift im ISSN-Portal möglich?
*Leichtes Identifizieren bzw. Kontaktieren der Verlegerin oder des Verlegers der Zeitschrift möglich?*
*Werden Anzahl der externen unabhängigen Gutachterinnen und Gutachter pro Arbeit sowie Art des Peer-Reviews und der Begutachtung *(durch fachkundigen Redaktionsausschuss oder durch Wissenschaftlerinnen und Wissenschaftler aus bestimmten Fachgebieten) *angegeben?*
*Werden die Artikel indexiert und/oder in speziellen Datenbanken archiviert?*
Verwendung dauerhafter digitaler Identifikatoren?
Sicherstellung der langfristigen Archivierung und Bewahrung digitaler Veröffentlichungen durch den Verlag?
*Werden anfallende Gebühren und mögliche Befreiungen genannt?*
Wird die Art der finanziellen Unterstützung des Verlags angegeben?
*Werden auf der Verlagswebseite Leitlinien für Autorinnen und Autoren bereitgestellt?*
Klare Lizenzvereinbarungen bei Open-Access-Zeitschriften?
Ist das Behalten des Urheberrechts an der eigenen Arbeit erlaubt?
Gibt es klare Vereinbarungen in Bezug auf Interessenkonflikte seitens der Autorinnen/Autoren, Redaktion und Gutachtenden?
*Ist der Verlag derzeit Mitglied einer anerkannten Brancheninitiative?*
Ist der Verlag derzeit Mitglied des Committee on Publication Ethics (COPE) und befolgt er dessen Richtlinien?
Ist die Zeitschrift frei zugänglich und im Directory of Open Access Journals (DOAJ) aufgeführt?
Ist der Verlag Mitglied des Open-Access-Verbands der Wissenschaftsverlage (OASPA)?

## Hilfestellungen und alternative Publikationsmodelle

Es gibt mittlerweile zahlreiche Hilfen im Internet, um Predatory Publishers, Journals und zweifelhafte Publikationspraktiken zu erkennen, allerdings gilt es auch hier, Vorsicht walten zu lassen, da einige der im Internet angebotenen Übersichten und Listen inzwischen selbst als räuberisch gelten. Andere lange Zeit als hilfreiche Ressource genutzte „blacklists“, wie z. B. die „Beall’s list“, wurden aufgrund zunehmender Kritik und angedrohter persönlicher Konsequenzen für den Betreiber stillgelegt.

Es ist nicht immer einfach, Predatory Journals zu erkennen, da ihre Webseiten denen etablierter Verlage oft ähneln und Vertrauenswürdigkeit suggerieren sollen. Informationen zu Publikationsgebühren und Reviewzeiten sind oftmals versteckt und schwer zu finden. Es gibt aber auch alternative Publikationsmodelle wie z. B. das DEAL-Abkommen oder verschiedene seriöse Open-Access-Journale.

Als vertrauenswürdige Hilfestellungen gelten zum jetzigen Zeitpunkt u. a.:

### Think. Check. Submit.

Think. Check. Submit. ist eine Initiative, die Forschenden helfen möchte, seriöse Journale auszuwählen. Sie bietet eine Checkliste, um die Qualität und Glaubwürdigkeit eines Journals zu überprüfen (Link: https://thinkchecksubmit.org).

### *Directory of Open Access Journals.*

Das Directory of Open Access Journals (DOAJ) ist ein Verzeichnis von Open-Access-Journalen, das nur Journale mit einem vertrauenswürdigen Peer-Review-Verfahren aufnimmt (Link: https://doaj.org).

### *DEAL.*

DEAL ist ein Konsortium zur Übernahme von Open-Access-Gebühren für die Angehörigen von deutschen Hochschulen bei einigen großen Verlagen (https://deal-konsortium.de/en/).

### *Journal Citation Reports.*

Der Journal Citation Reports (JCR) analysiert Zitationsdaten und bietet Impact-Faktor-Informationen für seriöse Journale. Hier lassen sich tatsächliche Impact Factors überprüfen (Link: https://jcr.clarivate.com/).

### *Cabells Scholarly Analytics’ Blacklist.*

Die Cabells Scholarly Analytics’ Blacklist bietet eine Übersicht (https://scholarlykitchen.sspnet.org/2017/07/25/cabells-new-predatory-journal-blacklist-review/).

**Abb. 1:**
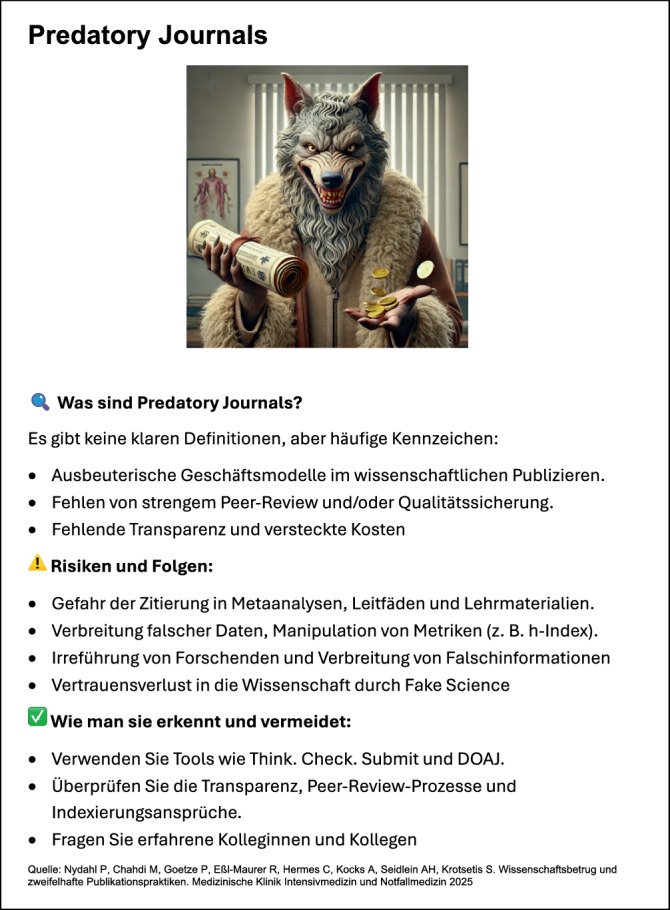
One Minute Wonder. (Quelle: Autorinnen und Autoren)

Wir empfehlen daher, bekannte und in PubMed und weiteren fachspezifischen Datenbanken, wie Cinahl, Scopus usw., gelistete Journale zu nutzen, wie beispielsweise *Medizinische Klinik, Intensivmedizin und Notfallmedizin*, und den teilweise langwierigen, aber qualitativ hochwertigen Weg des Peer-Reviews zu beschreiten. Mitarbeitende von Hochschulen in der Europäischen Union können zusätzlich von dem DEAL-Abkommen mit vielen anerkannten Verlagen profitieren, die die Übernahme von Open Access Gebühren ermöglicht. Wir haben dazu ein One Minute Wonder generiert (Abb. 1). Weiterhin empfehlen wir gerade im Hinblick auf die Karriereplanung, vor Einreichung bei einem Journal bei der eigenen Universität nachzufragen, welche Journale als relevant für die wissenschaftliche Arbeit anerkannt werden.

Neben der Publikationen in Predatory Journals lassen sich noch weitere Formen des Wissenschaftsbetrugs hervorheben.

## Weitere Formen des Wissenschaftsbetrugs

Zu dem Phänomen des Wissenschaftsbetruges gehören neben Predatory Publishers und Journals auch „paper“, „review“ und „citations mills“, die unethische Netzwerke darstellen und den wissenschaftlichen Publikationsprozess manipulieren [6]. In einer Analyse von 416 zurückgezogenen Publikationen im Jahr 2022 kamen über 90 % dieser Publikationen aus 3 „paper mills“ aus China, die zu > 40 % einander zitierten [6]:

- *„Paper mills“* verkaufen vorgefertigte oder gefälschte wissenschaftliche Artikel, oft mit erfundenen Daten, oder bieten „Autorenplätze“ auf Artikeln an. Sie kooperieren häufig mit Predatory Journals, um die Veröffentlichung zu sichern.
- *„Review mills“ *erstellen gefälschte Peer-Review-Berichte, um Artikel ohne Qualitätsprüfung zu veröffentlichen. Sie arbeiten mit unseriösen Journalen zusammen, die ein legitimes Begutachtungsverfahren vortäuschen.
- *„Citation mills“* erhöhen gegen Bezahlung Zitationszahlen von Artikeln oder Autorinnen und Autoren, oft durch künstliche Netzwerke gegenseitiger Zitationen.

Diese Praktiken gefährden die Glaubwürdigkeit der Wissenschaft, verfälschen Rankings, wie den h‑Index, und verbreiten falsche Forschungsergebnisse [3, 14]. Institutionen und Journale reagieren mit Rückrufen, strengeren Kontrollen und Transparenzinitiativen, um die Integrität des Publikationssystems zu schützen [7]. Hinweis: Auf https://retractionwatch.com/ werden zurückgezogene Manuskripte gelistet und oftmals Hintergrundinformationen bereitgestellt. Problematisch ist, dass diese zurückgezogenen Publikationen dennoch einige Zeit verfügbar sind und in Metaanalysen eingeschlossen werden können, die Autorenschaft der Metaanalysen über die spätere Zurückziehung einer eingeschlossenen Studie nicht informiert wird und der Ausschluss aber in der Metaanalyse zu signifikant anderen Ergebnissen führen kann [8]. Im Prinzip werden dadurch vernetzte, interaktive und kommunizierende Bibliotheken und Datenbanken erforderlich, die am ehesten durch künstliche Intelligenz organisiert werden.

Eine weitere Gefahr stellen Einladungen zu Fake Conferences dar.

## „Dear Professor, due to your prestigious publication about something we invite to a conference in Dubai“

Firmen, die Predatory Journale führen, organisieren oftmals auch Predatory Conferences [13]. Sobald der eigene Name, Titel der Veröffentlichung und E‑Mail-Adresse bei PubMed gelistet wurden, können Firmen mit Suchprogrammen diese Informationen sammeln und individuell formulierte Anschreiben und Einladungen versenden. Diese beginnen typischerweise mit „Dear Professor“ oder „Dear Doctor“ – unabhängig davon, ob die angeschriebene Person diesen Titel trägt. Als Begründung für die Einladung wird eine frühere Publikation der Person zitiert gefolgt von der Einladung zu einer Konferenz im Ausland. Für diese Konferenzen existieren zwar Webseiten, die aber nur der Täuschung dienen. Derartiger akademischer Spam verunsichert viele Forschende, die diesen erst mit wachsender Erfahrung als solchen erkennen [13]. Die Konferenzen sind kostenpflichtig und meist eine Sammlung unterschiedlichster Disziplinen. Es lohnt sich, hier die 30-minütige ARD-Dokumentation „Fake Science – Die Lügenmacher“ zu sehen (Link: https://www.ardmediathek.de).

Wir empfehlen, solche Einladungen kritisch zu prüfen, bei Unsicherheiten mit erfahrenen Kolleginnen und Kollegen in den Austausch zu gehen und nur auf bekannte Angebote und Personen zu reagieren. Häufig bieten auch Fachreferentinnen und Fachreferenten sowie Mitarbeitende in Bibliotheken mit ihrem umfangreichen Wissen Unterstützung und Beratung an.

Neben Einladungen zu Fake Conferences kann ein weiteres Phänomen auftreten: die Einladung, Fake-Manuskripte zu reviewen.

## Fake-Manuskripte reviewen und erkennen

Mitunter erhalten Wissenschaftlerinnen und Wissenschaftler die Gelegenheit, internationale Manuskripte zu begutachten. Als Reviewerinnen und Reviewer ist uns hier eine zunehmende Entwicklung von Ungereimtheiten in englischsprachigen Manuskripten aufgefallen, die wir noch nicht bewerten können, aber die wir anekdotisch berichten möchten. Zunächst hat sich seit der Veröffentlichung von ChatGPT die Sprachqualität von Manuskripten aus China sprunghaft verbessert, ohne dass das Nutzen der KI in den Manuskripten berichtet worden ist. Weiter fallen in den letzten Jahren zunehmend Manuskripte aus China, der Türkei und auch dem Iran auf, bei denen es sich scheinbar um perfekte randomisierte, kontrollierte Studien handelt: Die Manuskripte sind exzellent geschrieben, enthalten alle erforderlichen Beschreibungen und Kriterien, die eingeschlossene Populationsgröße liegt stets mit 3–5 Personen über der angestrebten Power-Kalkulation, die Interventionsgruppe ist oft etwas kränker als die Kontrollgruppe (APACHE-II-Score, Alter, Komorbiditäten), aber ohne statistische Signifikanz. Weiter gibt es eine geringe bis keine Dropout-Rate, das primäre Outcome ist signifikant verbessert und von den sekundären Outcomeparametern sind in den Gruppen nie 100 %, aber 80 % signifikant unterschiedlich. Aber es werden keine Interventionsdaten berichtet, d. h.: Es ist unbekannt, wie viele Patientinnen und Patienten die Intervention tatsächlich oder nur teilweise erhalten haben und wie die Effektgrößen dabei sind. Dies wirft Zweifel an der wissenschaftlichen Integrität der Forschenden hinter diesen Arbeiten auf, die damit nicht reliabel oder vertrauenswürdig zu sein scheinen, was zu der Empfehlung der Ablehnung führt.

Interessanterweise kann auch hier ein Lernprozess beobachtet werden: So konnten vor 5 Jahren in fast jedem Manuskript die fehlenden Qualitätskriterien, wie die von www.equator-network.org, oder bei Übersichtsarbeiten die fehlende Registrierung in Prospero (www.crd.york.ac.uk/prospero/) bemängelt werden, in den heutigen Manuskripten sind diese Informationen meistens enthalten. Wir sind gespannt, ob in zukünftigen RCT die fehlenden Interventionsdaten also enthalten sein werden, was ein Review sehr schwierig machen wird, denn natürlich können auch echte RCT darin enthalten sein.

## Wissenschaftsbetrug und zweifelhafte Publikationspraktiken erkennen!

Predatory Journals untergraben die ethischen Prinzipien der Wissenschaft, denen auch die Publikationsprozesse folgen (sollten; vgl. COPE https://publicationethics.org/ oder Deutsche Forschungsgemeinschaft: https://zenodo.org/records/6472827).

## Schlusswort

Dieser Artikel zeigt nicht nur unlautere und gegen bestehende ethische Grundsätze der Wissenschaft agierende Geschäftsmodelle auf, sondern auch, wie dadurch die eigene Forschungsarbeit manipuliert und die Qualität wissenschaftlicher Empfehlungen gefährdet und letztlich das Vertrauen in die Wissenschaft zerstört werden kann.

Wissenschaftlerinnen und Wissenschaftler sollten in einer zunehmend digitalisierten, schnelllebigen und durch soziale Medien geprägten Welt der Nachrichten sich die Zeit nehmen zu schauen, wem sie ihre seriöse Arbeit und ihren guten Namen anvertrauen wollen. Vor dem Hintergrund des Diktums „publish or perish“ sollten sie daher auch beherzigen: „take care in whom you trust“.
